# A rapid analysis of Bisphenol A using MISPE coupled with HPLC-FLD in tissues of food-producing animals

**DOI:** 10.1016/j.mex.2021.101351

**Published:** 2021-04-18

**Authors:** Vesna Cerkvenik-Flajs, Sabina Šturm

**Affiliations:** University of Ljubljana, Veterinary Faculty, Institute of Pathology, Wild Animals, Fish and Bees, Gerbičeva 60, SI-1000 Ljubljana, Slovenia

**Keywords:** Bisphenol A, Tissue analysis, Solid phase extraction, Molecularly imprinted polymers, Liquid chromatography

## Abstract

Bisphenol A (BPA) is a highly-produced organic compound of anthropogenic origin with a wide-range use and is ubiquitously present in both living organisms and the environment. A previously published analytical method for testing of the free (aglycone) BPA in foodstuffs was simplified and optimized for sheep muscle tissue, kidney and liver, by using only a single MISPE purification step allied with HPLC-FLD analysis. The recovery rates and RSD values over a concentration range of 1–10 µg/kg were in the range of 67‒86% and 3‒34%, respectively, while linearity in the matrix, represented by the r^2^, was ≥0.999. LOD values were 0.5‒1 µg/kg and LOQ values were 1 µg/kg. The analytical method used is a contribution to the field of veterinary toxicology and food testing and also proved to be applicable for other food-producing animal species, e.g. pigs, poultry and freshwater fish. The MISPE sorbent material for testing of BPA was reusable, with up to five re-use cycles without significant loss in performance characteristics.

• The paper reports on a rapid determination of free (aglycone) BPA in tissues of food-producing animals by HPLC-FLD and presents a substantial improvement of analytical performance with regard to time- and cost-savings, as well as environmental protection.
• MISPE is an advanced analytical technology, and the results proved that in a single and simplified SPE step it had good performance characteristics such as selectivity, recovery and precision, allied with low LOD and LOQ values.
• It was proved that MISPE BPA cartridges could be used at least five times without significant loss of methodological recovery and precision.

## Graphical abstract

**Figure:**
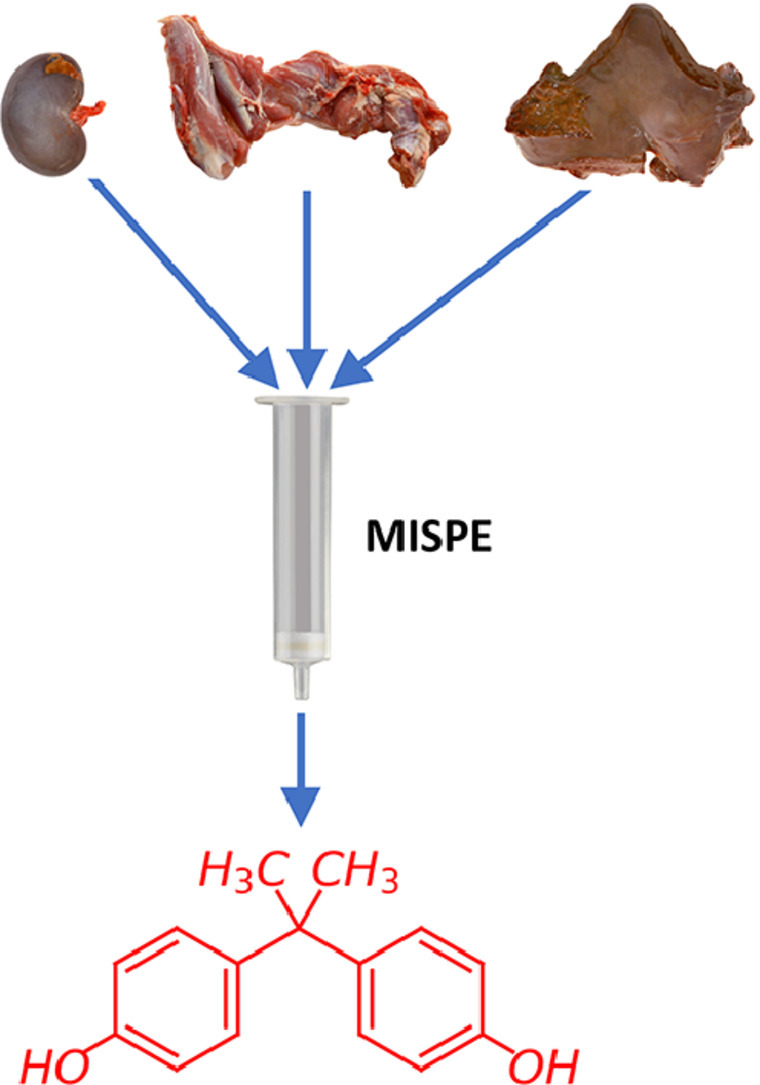

Specifications table

**Table:** 

Subject area:	Veterinary Science and Veterinary Medicine
More specific subject area:	*Veterinary Toxicology and Food Control*
Method name:	*Analysis of BPA using MISPE coupled with HPLC-FLD in tissues of food-producing animals*
Name and reference of original method:	*Development and validation of a specific and sensitive gas chromatography tandem mass spectrometry method for the determination of bisphenol A residues in a large set of food items. Deceuninck Y, Bichon E, Durand S, Bemrah N, Zendong Z, Morvan ML, Marchand P, Dervilly-Pinel G, Antignac JP, Leblanc JC, Le Bizec B. J Chromatogr A 2014;1362:241–49*
Resource availability:	doi:10.1016/j.chroma.2014.07.105

## Method details

Bisphenol A (BPA; CAS registry no. 80-05-7, synonyms 2,2- bis(4-hydroxyphenyl)propane, 4,4′-isopropylidenediphenol) is an organic synthetic compound, currently produced in enormous quantities throughout the world. It is primarily used as a precursor in the production of polycarbonate plastics and epoxy resins. The global market for BPA is projected to reach 7.1 million tons by the year 2027 [1]. Exposure to environmental nanomolar concentrations of BPA is ubiquitous and continuous. BPA has been shown to exert endocrine-disrupting effects and interferences with the developmental processes of humans and animal species [2]. Recent investigations refer to toxic, teratogenic, carcinogenic and particularly estrogenic mechanisms of free BPA action [3]. Low doses of BPA that are environmentally relevant might play a role in the development of chronic metabolic diseases, reproductive pathologies, hormone dependent cancers (prostate, breast, testes). High levels of BPA have recently been correlated with obesity, diabetes, cardiovascular diseases, polycystic ovarian disease and low sperm count [3,4]. In 2015, the European Food Safety Authority (EFSA) reduced the tolerable daily intake (TDI) for BPA from 50 to 4 µg/kg bw/day [5]. The European Chemicals Agency (ECHA) has added BPA to the Candidate List of New Substances of Very High Concern, causing adverse effects to the environment [6].

Many analytical methods for the detection of BPA in environmental and biological materials have been developed, including chromatography and mass spectrometry-based methods, and some novel methods such as sensors. Nevertheless, sample pretreatment is a key step for the detection of BPA, but most sample preparations are time-consuming and labour-intensive [7]. Since 2000, sample pretreatment methods have undergone considerable development, also due to the use of nano-structured liquids, named supramolecular solvents that combine extraction and clean-up in a single stage [8,9], and due to selective sorbents, such as molecularly imprinted polymers (MIPs), which are synthetic tailor-made separation polymers having a predetermined selectivity for a given analyte, or group of structurally related compounds. MIPs are obtained by polymerizing functional and cross-linking monomers around a template molecule, leading to a highly cross-linked three-dimensional network polymer [10,11]. The resulting imprinted polymers are highly stable, robust and resistant against physicochemical perturbations such as pH value, solvents and temperature. Therefore, the technique of molecular imprinting provides a promising and advantageous alternative to overcome the problems associated with biomolecules such as antibodies, enzymes, and other receptor molecules [12]. MIPs have the potential to enable analyte isolation and clean-up in a single-step and provide cleaner extracts compared to, for example, ”classical‟ SPE [13].

Nowadays, the use of MIPs in solid phase extraction, so-called molecularly imprinted solid-phase extraction (MISPE), is by far the most advanced technical application of MIPs. A review of the analytical methods for detection of BPA by Sun et al. showed that the majority of the matrices investigated were water, sediments and biofluids (urine, milk) [7]. Regarding tissue analysis, two analytical methods, by Wei et al. using a high-performance liquid chromatography (HPLC) coupled to fluorescence detection (HPLC–FLD) [14], and by Di Marco Pisciottano et al. using liquid chromatography coupled to tandem mass spectrometry (LC–MS/MS) [15], were reported for MISPE determination of BPA in fish samples. To the best of our knowledge, only the method of Deceuninck et al. comprised using MISPE for determination of free (aglycone) BPA in meat and offal using gas chromatography coupled with tandem mass spectrometry (GC–MS/MS) [16]. This method was used for assessment of dietary exposure to BPA in the French population, by Bemrah et al., where the levels of BPA found in meat, poultry and game, and offal were in the range of 0.1–224, 0.4–49 and 0.6–395 µg/kg, respectively [17].

The objectives of the present study were to systematically optimize the critical factors affecting the SPE step within the free BPA tissue determination in the edible tissues of sheep (i.e. muscle, kidney and liver). Within our recent research on BPA we were faced with relatively weak recovery rates for the analysis of marine mussel tissue [18] and sheep faeces [19] with a mean value of 47 and 52%, respectively. The clean-up of both analytical methods used was based on the method of Deceuninck et al. [16], by omitting the derivatization step and using HPLC-FLD instead of GC-MS/MS. Moreover, on the basis of the reported reusability of the MIP by Gallego-Gallegos et al. [11] and a recent publication on the successful multiple use of MIP cartridges for determination of ochratoxin A in pig muscle, kidney and liver by Luci [20], we also evaluated the possible multiple use of MISPE BPA cartridges for testing the sheep matrices investigated in the current work.

## Materials and methods

### Reference standards

The certified reference standard of BPA was obtained as a powder of 99.0% analytical purity from Sigma-Aldrich (Merck, Darmstadt, Germany). The stock standard solution of 200 µg/ml was prepared in acetonitrile (MeCN) and kept frozen (at −20 °C), while the intermediate and working standard solutions ranging from 100 to 2,000 ng/ml were further prepared in a mixture of MeCN/H_2_O (35/65, v/v) and kept refrigerated (at 4–8 °C). Calibration standards of ≤50 ng/ml were prepared on a daily basis.

### Reagents and consumables

The MeCN and methanol (MeOH) used, which were HPLC gradient-grade purity, were purchased from J. T. Baker (Center Valley, PA, USA). The high purity deionized water used with a resistivity of 18.2 MΩ.cm was obtained by a PureLab Option and PureLab Classic water purification system (Elga, Woodridge, Illinois, USA). The formic acid (HCOOH), 98–100% of reagent grade purity, was supplied by Merck (Darmstadt. Germany). The solid-phase extraction (SPE) columns used were Chromabond HR-X, 6 mL, PP, with an 85 µm particle size and 200 mg of sorbent, which were supplied by Macherey-Nagel (Düren, Germany), and MIP AFFINIMIP® SPE Bisphenols, 6 ml, with 100 mg of sorbent, which were supplied by AFFINISEP (Petit Couronne, France). The centrifuge tubes (15 ml, conical, screw cap, PP) were supplied by Isolab (Wertheim, Germany), and the centrifuge tubes (15 ml, conical, glass) were supplied by Brand (Wertheim, Germany). The dark glass amber, 1.5 ml vials were obtained from La−Pha−Pack (Langerweche, Germany).

### Equipment

Tissue samples were homogenized using a mini chopper (Russell Hobbs, Manchester, UK). An electronic balance Vibra AJ ‒ CE/AJH ‒ CE (± 0.001 g) and a Vibromix 10 were obtained from Domel (Železniki, Slovenia), a Transsonic 460/H ultrasonic bath was acquired from Elma (Singen, Germany), and a centrifuge Centric 350 was procured from Domel (Železniki, Slovenia). An SPE Vacuum Manifold Visiprep 24 was supplied by Sigma-Aldrich (Merck, Darmstadt, Germany) and an N-EVAP 111 evaporator was provided by Organomation Associates (Berlin, MA, USA). The HPLC system used was a Varian ProStar (Varian Analytical Instruments, Walnut Creek, CA, USA), which comprised a tertiary pump (240 model), an automatic injector (410 model), a fluorescence detector (363 model), a degasser, and Galaxie 1.7.4.5 analytical software.

### Sample extraction

An aliquot of 2.0 ± 0.005 g of a homogenized (defrosted) sample was weighed into a 15 ml plastic (PP) centrifuge tube and extracted with 8 ml of MeCN by vortexing for 2 min and ultrasonication for 13 min. After repeated vibromixing for 1 min, the sample was centrifuged at room temperature for 10 min at 2640 × g and re-extracted using 2 ml of MeCN. The combined supernatants were then evaporated under a N_2_ stream at 40 °C to an aqueous residue. When performing the original method, a sample residue was dissolved using 0.3 ml of MeOH and 4.7 ml of H_2_O, while when performing a present method, it was dissolved using 1 ml of MeCN and 10 ml of H_2_O, before applying onto the SPE cartridge.

### Solid-phase extraction (SPE) by the original method [16]

The workflow is presented in Table 1. The sample extract was applied under gravity onto the SPE Chromabond HR-X cartridge, which had previously been conditioned with 10 ml of MeOH and 10 ml of H_2_O. The column was washed with 6 ml of H_2_O, 8 ml of MeOH/H_2_O (10/90, v/v) and 4 ml of MeOH/H_2_O (60/40, v/v). Elution was performed under gravity with 10 ml of MeOH into a 15 ml glass centrifuge tube, and the SPE eluate was evaporated under a N_2_ stream at 40 °C just to dryness. The remains were re-dissolved using 0.2 ml of MeCN and 5 ml of H_2_O, before applying under gravity onto the second SPE – MIP BPA cartridge, being conditioned with 10 ml of MeOH/HCOOH (98/2, v/v), 4 ml of MeCN and 4 ml of H_2_O. The column was washed with 5 ml of H_2_O, 3 ml of MeCN/H_2_O (40/60, v/v) and 3 ml of MeCN. After drying the sorbent under a vacuum, the BPA was eluted under gravity with 4 ml of MeOH (modification in relation to [16], using 6 ml of MeOH) into a 15 ml glass centrifuge tube, and the SPE eluate was evaporated under a N_2_ stream at 40 °C just to dryness. Final sample extracts were re-dissolved in 0.8 ml of MeCN/H_2_O (35/65, v/v), ultrasonicated for 5 min, vibromixed, centrifuged at room temperature for 10 min at 2640 × g, and transferred into a HPLC vial.

**Table 1:** Structure of SPE steps for determination of free BPA in edible tissues using the original method of Deceuninck et al. [16] and by the one proposed in the present study.

SPE step	Phase	Extraction protocol	
		*Original method of Deceuninck et al.**[16]*	*Present study*
**HR-X stationary phase**		included	skipped
**MISPE BPA stationary phase**	***Conditioning the cartridge***	10 ml MeOH/HCOOH (98/2, v/v) 4 ml MeCN 4 ml H_2_O	10 ml MeOH/HCOOH (98/2, v/v) 4 ml MeCN 4 ml H_2_O
	***Loading the extract***	diluted in 0.2 ml of MeCN and 5 ml of H_2_O	diluted in 1 ml of MeCN and 10 ml of H_2_O
	***Washing***	5 ml H_2_O 3 ml H_2_O/MeCN (60/40, v/v) 3 ml MeCN	5 ml H_2_O 3 ml H_2_O/MeCN (60/40, v/v)
	***Elution***	4 ml MeOH (modification)	6 ml MeOH

### Solid-phase extraction (SPE) by the present method

The sample extract was applied under gravity onto the MIP BPA cartridge (Table 1), being conditioned with 10 ml of MeOH/HCOOH (98/2, v/v), 4 ml of MeCN and 4 ml of H_2_O. The column was washed with 5 ml of H_2_O and 3 ml of MeCN/H_2_O (40/60, v/v). After drying the sorbent under a vacuum, the BPA was eluted under gravity with 6 ml of MeOH into a 15 ml glass centrifuge tube, and the SPE eluate was evaporated under a N_2_ stream at 40 °C just to dryness. Final sample extracts were re-dissolved in 0.8 ml of MeCN/H_2_O (35/65, v/v), ultrasonicated for 5 min, vibromixed, centrifuged at room temperature for 10 min at 2640 × g, and transferred into a HPLC vial.

### HPLC-FLD analysis

A 50 µl aliquot of the final sample extract was taken for the HPLC analysis. A Hypersil GOLD C18 (150 × 4.6 mm, 3 μm particle size) analytical column was used which was protected by a Hypersil GOLD 3μ drop in guard cartridges (Thermo Scientific, Waltham, MA, USA). The chromatographic process was performed at room temperature (25‒26 °C), while the mobile phase as a mixture of H_2_O (constituent A) and MeCN (constituent B) was pumped at a flow rate of 1.0 ml/min and the excitation and emission wavelengths of the fluorescence spectrometric detection were set at 230 and 315 nm, respectively [21]. For muscle tissue and kidney analysis, the mobile phase constituents were gradient pumped in the following volume ratios: time 0–2 min (35% B), time 2–12 min (gradient 35–50% B), time 12–20 min (50% B), time 20–20.5 min (gradient 50–35% B), time 20.5–21 min (35% B) [21]. For liver analysis, the last gradient ramp (50–35% B) was prolonged from 20–25 min, and thus the whole chromatographic run time for this matrix was 25.5 min. BPA sample concentrations were calculated according to the external standard calibration.

### Method validation

The analytical methodology was validated to demonstrate its fitness for determination of the BPA in the edible tissues of sheep. Selectivity was evaluated by an appropriate number of representative baseline samples of sheep (*n* = 11‒16 per matrix) being analysed and checked for any peak interferences in the retention time region of BPA. Linearity was determined on both standard and matrix levels by the least squares method, giving the regression and correlation parameters of the calibration lines. Solvent standard concentrations ranged from 0.5–250 ng/ml with 4–8 concentration points per calibration line. Linearity in the matrix, recovery, and precision of BPA determination in the sheep muscle tissue, kidney and liver were evaluated by fortification of a baseline sample, being provided by the Infrastructure Centre for Sustainable Recultivation Vremščica of the Veterinary Faculty of the University of Ljubljana and also obtained from the city market of Ljubljana. Linearity on a matrix level was evaluated as an intra-day correlation between the mean measured and added BPA concentrations (*n* = 2/fortification level over a concentration range of 0.5–100 µg/kg and 1–100 µg/kg for muscle tissue and kidney, and liver, respectively). Recovery, repeatability and intra-laboratory reproducibility were tested on three BPA concentration levels of 1, 5 and 10 µg BPA/kg. Repeatability was evaluated at the same time, while intra-laboratory reproducibility was evaluated at the two separate times. The precision of the methods was evaluated using the standard deviation (SD) and the relative standard deviation (RSD) of the determined values, and assessed in accordance with the Horwitz coefficients (RSD_H_) according to the European Commission Decision 2002/657/EC [22]. Matrix effect was estimated by fortification of a baseline matrix final extract (*n* = 2/concentration level) with the BPA at concentration, representing 20, 10 and 5 µg/kg in the tissue sample, and comparison of a chromatographic response with those of equivalent solvent standard. The limit of detection (LOD) value was estimated as the minimum detectable amount of BPA from matrix samples with a signal-to-noise ratio of 3:1, and was corrected for the baseline matrix response, while the limit of quantification (LOQ) value was determined as the lowest analyte content for which the analytical method proved acceptable in terms of recovery and repeatability [23]. The method's suitability for use regardless of the animal species was tested as follows: five negative samples of sheep, chicken, pig and aquaculture muscle tissue (freshwater fish) were simultaneously analysed, each at a fortification level of 10 µg BPA/kg. Additionally, five negative samples of sheep, chicken and pig liver, and sheep and pig kidney, were simultaneously analysed, each at a fortification level of 10 µg BPA/kg. The recovery and repeatability of the measurements were evaluated.

### Reusability of MISPE BPA cartridges

MISPE BPA cartridges were used five times for simultaneous testing of two to five samples of sheep muscle tissue, kidney and liver, fortified at 10 µg BPA/kg. The efficiency of the MIP stationary phase with multiple use was evaluated based on the recovery achieved after individual use according to the matrix investigated.

## Results and discussion

### Dealing with samples

Homogenized tissue samples were stored in PP containers at −20 °C until analysis. The importance of appropriate quality control of sample testing was considered, as blank controls were included with each batch of the fortified samples analysed to avoid and minimize possible artifacts or contamination and ensure appropriate performance characteristics of the BPA analytical method [22]. In addition, storage devices with declared absence of BPA were used, high quality glassware was used where possible, and the solvents used were mainly of HPLC grade and screened via reagent blanks.

### Method development

In the present study the critical factors affecting the SPE step (nature of SPE stationary phase, nature of SPE washing solutions) within the free (aglycone) BPA tissue determination were optimized using fortified sheep tissue samples. Sheep tissues were chosen to establish the analytical conditions for testing materials obtained from a toxicokinetic and reproductive toxicity BPA study in rams (unpublished data). Moreover, on the basis of the recent discovery about the successful reusability of MISPE cartridges for determination of ochratoxin A in pig muscle, kidney and liver [20], we also checked the possible multiple use of MISPE BPA cartridges for testing the matrices investigated.

**Fig. 1:**
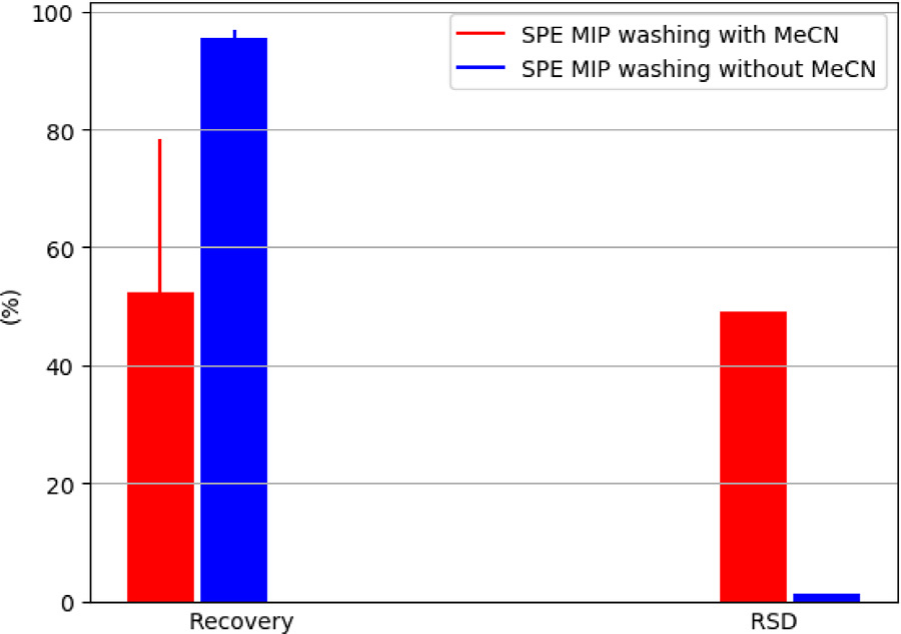
Optimization of MISPE BPA washing with vs. without usage of MeCN, represented by recovery ± SD and RSD; 3 replicates of fortified sheep muscle tissue at 10 µg BPA/kg per washing mode were used.

The use of SPE for sheep muscle tissue, kidney and liver, according to the original method of Deceuninck et al. [16], resulted in mean recoveries of BPA (fortification level 10‒20 µg/kg, *n* = 4) of 47, 24 and 28%, respectively. Simultaneous verification of the MISPE washing with vs. without usage of 4 ml MeCN, demonstrated enhanced recovery without usage of MeCN in sheep muscle tissue from 52.4 to 95.4% and lowered relative standard deviation (RSD) from 49.1 to 1.3%, respectively (fortification level of 10 µg BPA/kg, *n* = 3) (Fig. 1). Consequently, from this step onward, washing of MISPE BPA with MeCN was excluded from the analytical procedure to omit large and variable losses of bound BPA from the cartridge sorbent. This finding also demonstrated different MISPE BPA behaviours for clean-up of different matrix structures, e.g. for blood plasma [24] and urine [19], where recovery rates were significantly higher (>76 and >60%, respectively) after washing the cartridge with MeCN, compared to, for example, the tissue of mussels (recovery of 47%) [18]. The MISPE clean-up simplification was also systematically described for ochratoxin A determination in pig muscle tissue, kidney and liver [20].

**Fig. 2:**
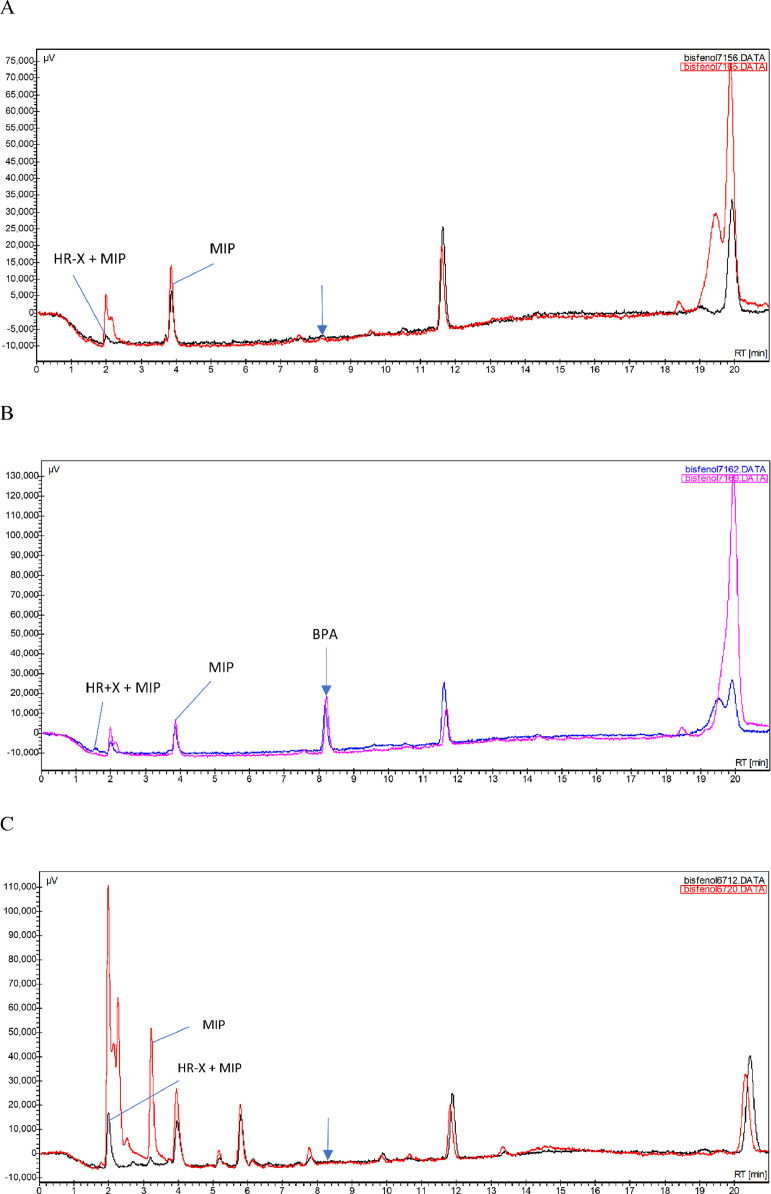

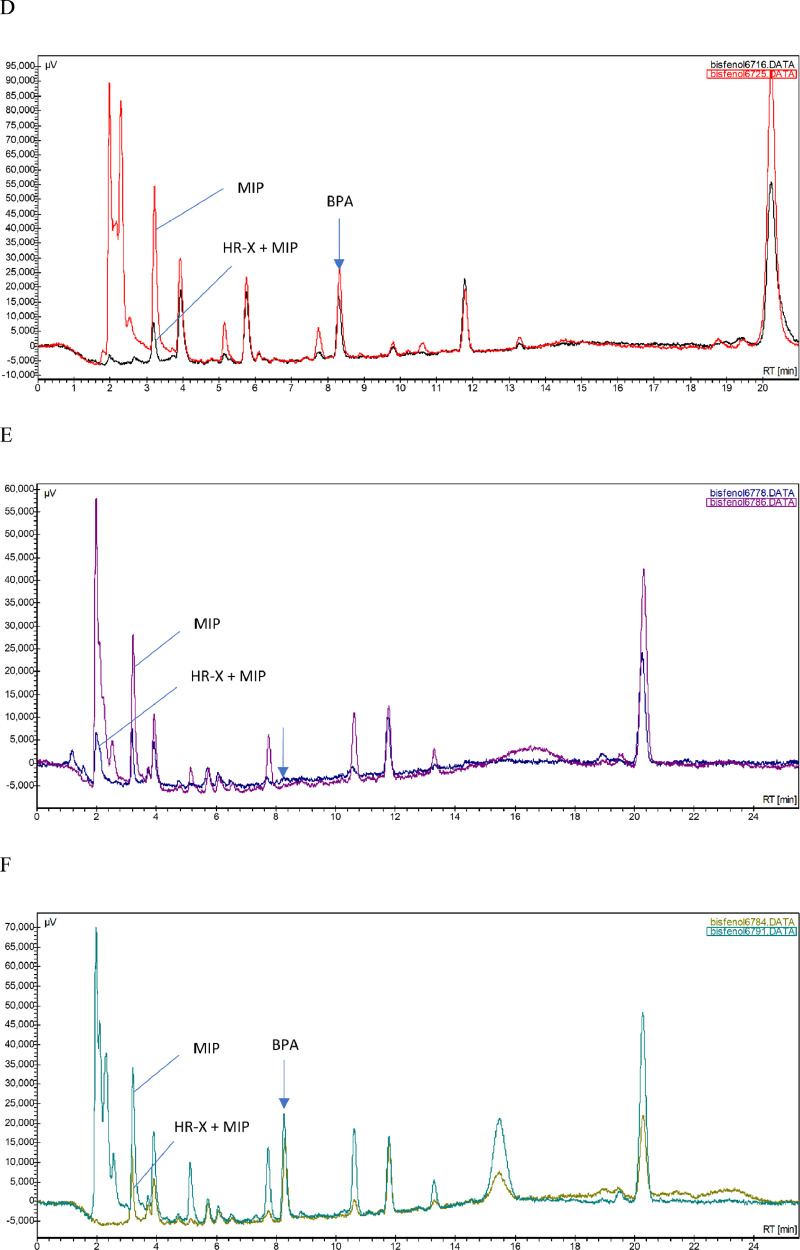
Representative HPLC chromatograms for the determination of the free bisphenol A (BPA) in sheep muscle tissue, kidney and liver, comparing the combined use of SPE HR-X and MISPE BPA cartridges vs. MISPE cartridges only: (A) muscle tissue, blank sample; (B) muscle tissue, blank sample fortified with 10 µg BPA/kg; (C) kidney, blank sample; (D) kidney, blank sample fortified with 10 µg BPA/kg; (E) liver, blank sample; (F) liver, blank sample fortified with 10 µg BPA/kg.

The second step aimed to simplify the clean-up procedure as much as possible with a hypothetical withdrawal of an SPE with HR-X stationary phase and using only the MISPE stationary phase [15]. A higher matrix background was revealed when using only a single SPE. Nevertheless, the selectivity of the chromatographic separation and FLD was sufficient, as demonstrated by Fig. 2. Liquid chromatography-fluorescence (LC-FLD) is due to its high specificity and selectivity considered a suitable confirmatory method for organic residues or contaminants (non-prohibited) that exhibit native fluorescence (such as BPA) and to molecules that exhibit fluorescence after either transformation or derivatisation [22].

Using only the MISPE BPA step instead of a combination of both SPE HR-X and MISPE BPA significantly improved recovery for sheep muscle tissue, kidney and liver, from 71 to 89%, from 62 to 86%, and from 60 to 79%, respectively. The RSD value of determination conversely decreased markedly for muscle tissue and liver, from 10 to 1% and from 14 to 2.5%, respectively, while for kidney a slight increase was observed, from 5 to 7% (Table 2).

**Table 2:** Optimization of SPE for BPA determination in edible tissues of sheep with regard to the cartridges' use; 3 replicates of fortified muscle tissue, kidney and liver (10 µg BPA/kg) per SPE mode were tested.

Matrix	Rec_mean_ (%)		SD (%)		RSD (%)	
	SPE HR-X + MIP	MISPE	SPE HR-X + MIP	MISPE	SPE HR-X + MIP	MISPE
Muscle tissue	70.8	89.1	7.0	1.1	10.0	1.3
Kidney	62.2	86.3	3.4	6.1	5.4	7.1
Liver	59.7	79.3	8.3	1.9	13.9	2.5

### Method validation

**Fig. 3:**
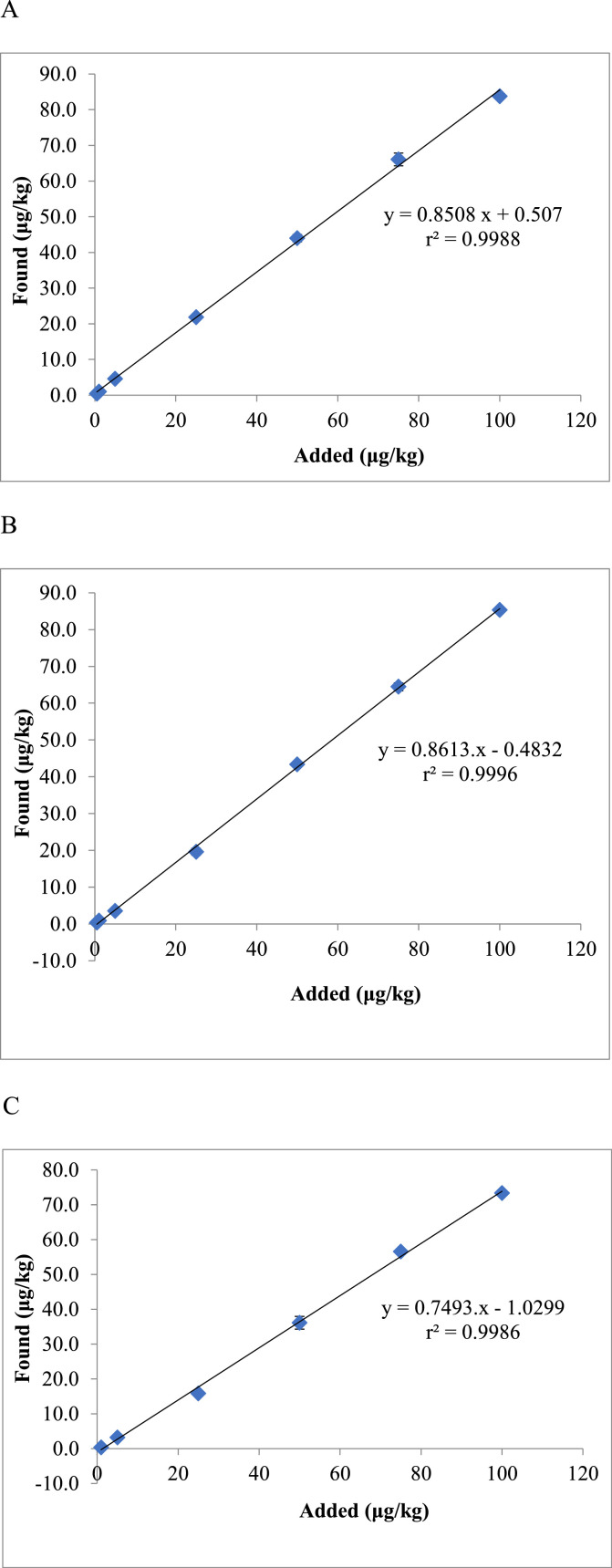
Linearity of analytical HPLC determining the level of free bisphenol A (BPA) in sheep matrices, evaluated as an intra-day correlation between the mean measured and added BPA concentrations (*n* = 2/fortification level); difference bars of both parallels are also presented: (A) muscle tissue, fortification range from 0.5–100 µg/kg (B) kidney, fortification range from 0.5–100 µg/kg (C) liver, fortification range from 1–100 µg/kg.

Very relevant analytical performance characteristics were obtained. Chromatograms obtained for baseline sheep's samples did not show any peak near the retention time of BPA as the response was <LOD for the matrices investigated. The linearity of the standard calibration curves expressed by the correlation coefficient was ≥0.9997 for the concentration range of 1–50 ng/ml, including 6 calibration points (*n* = 9) and ≥0.9999 for the concentration range 1–250 ng/ml, including 8 calibration points (*n* = 3). The linearity of determining the level of BPA in the matrices investigated is presented in Fig. 3, based on the correlation curves between the found and added values of analyte. The “r-squared” values were 0.9988, 0.9996 and 0.9986 for the sheep muscle tissue, kidney and liver, respectively.

Corresponding to concentration range from 20 to 5 µg BPA/kg there was a weak matrix effect from -0.9 to -1.9% for muscle tissue, while for kidney a decrease of a chromatographic peak area from -3.6 to -19% was observed in comparison with the solvent standards. A weak matrix effect from -0.9 to -1.9% was also observed for liver at a concentration range from 20 to 10 µg BPA/kg, while at concentration of 5 µg BPA/kg of liver an increase of a chromatographic peak area for 11% was observed in comparison with the solvent standards. The recovery and precision of the method are presented in Table 3 and were determined on three levels of content for each matrix. The recovery values for determination of BPA in muscle tissue, kidney and liver ranged from 78 to 86%, from 67 to 85%, and from 69 to 81%, respectively. The repeatability of the measurements, represented by the daily RSD values, ranged from 6.6 to 9.3%, from 2.1 to 10.8% and from 2.1 to 8.2%, respectively. The within-laboratory reproducibility, represented by the intr-day RSD values, ranged from 8.0 to 20.6%, from 3.0 to 34.2% and from 3.1 to 11.6%, respectively. The RSD values did not exceed the RSD_H_ values from the Horwitz equation [22]. Moreover, with the exception for RSD value at fortification level of 1 µg/kg in kidney at the within-laboratory reproducibility conditions, of which the explanation lies in both, a very low concentration level and also a matrix effect for the kidney tissue, they were below one fourth of the corresponding RSD_H_ values, and thus fully acceptable (Table 3). The estimated LODs were 0.5 µg/kg for sheep muscle tissue and kidney and 1 µg/kg for liver, while the LOQ values determined were 1 µg/kg for all three matrices investigated in this study. This is comparable to typical reported detection limits for BPA in foods using FLD, being in the range of 0.1–2 ng/ml and 1–5 µg/kg [13].

**Table 3:** Recovery and precision of determination of BPA in sheep muscle tissue, kidney and liver.

Matrix	Fortification level (µg/kg)	Repeatability (*n* = 5)			Within-laboratory reproducibility (*n* = 10)			aRSD_H_ (%)
		Rec_mean_ (%)	SD (%)	RSD (%)	Rec_mean_ (%)	SD (%)	RSD (%)	
Muscle tissue	1	83	5.5	6.6	82	16.9	20.6	45
	5	86	7.1	8.2	86	6.9	8.0	36
	10	81	7.5	9.3	78	7.0	8.9	32
Kidney	1	85	9.2	10.8	67	22,9	34.2	45
	5	81	1.7	2.1	82	2.5	3.0	36
	10	85	3.2	3.7	84	2.7	3.2	32
Liver	1	74	6.1	8.2	69	8.1	11.6	45
	5	81	1.7	2.1	80	2.5	3.1	36
	10	78	2.7	3.5	78	3.0	3.9	32

a Horwitz coefficient of variation [22]

**Fig. 4:**
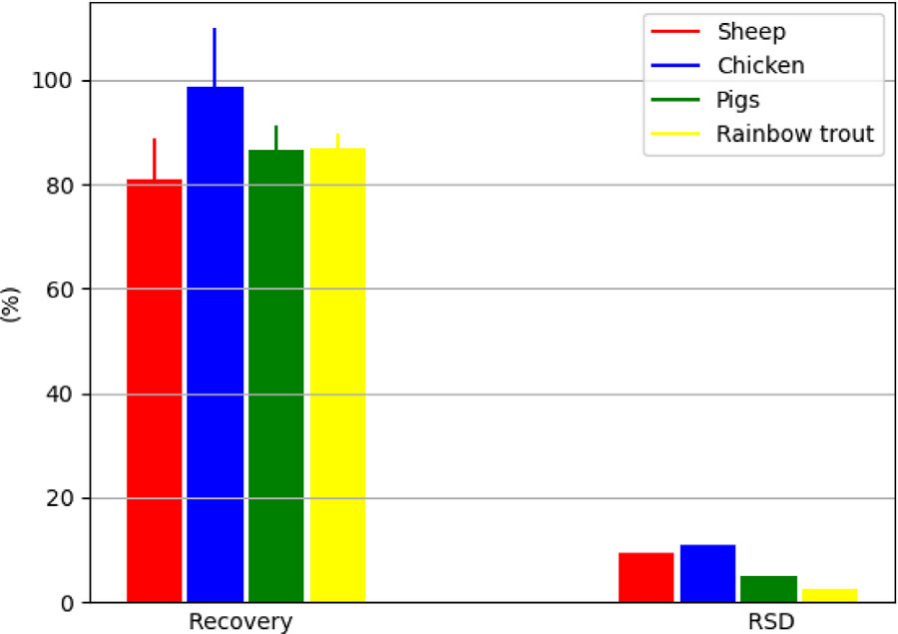
Determination of BPA in muscle tissue of different animal species, represented by recovery ± SD and RSD (*n* = 5 replicates/species at fortification of 10 µg/kg); in the case of rainbow trout (*Oncorhynchus mykiss*), muscle tissue with skin in natural proportion was taken for the analysis.

**Fig. 5:**
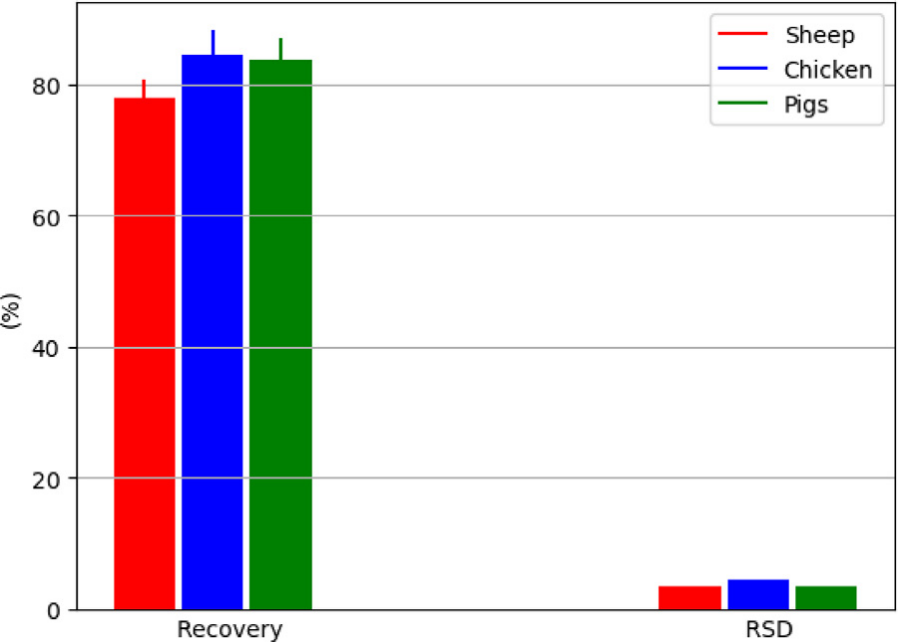
Determination of BPA in liver of different animal species, represented by recovery ± SD and RSD (*n* = 5 replicates/species at fortification of 10 µg/kg).

**Fig. 6:**
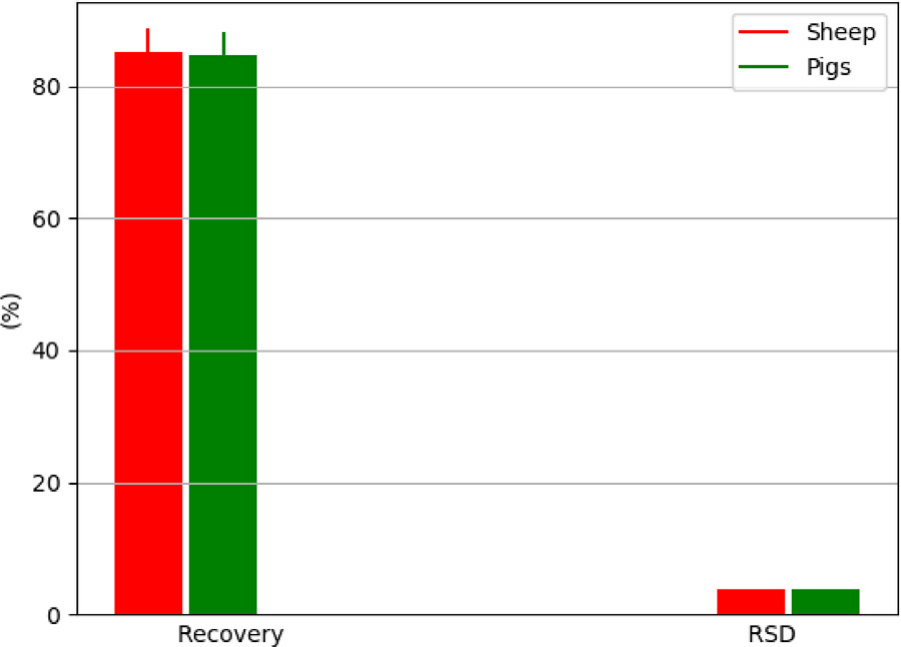
Determination of BPA in kidney of sheep and pigs, represented by recovery ± SD and RSD (*n* = 5 replicates/species at fortification of 10 µg/kg).

The method presented in this study proved to be applicable for use in various animal species, such as for the muscle tissue of sheep, chicken, pigs and freshwater fish (Fig. 4), for the liver of sheep, chicken and pigs (Fig. 5), and for the kidney of sheep and pigs (Fig. 6). This proved its general applicability in edible tissues of various food-producing animals, namely ruminants, poultry, pigs and aquaculture.

### Reusability of MISPE BPA cartridges

**Fig. 7:**
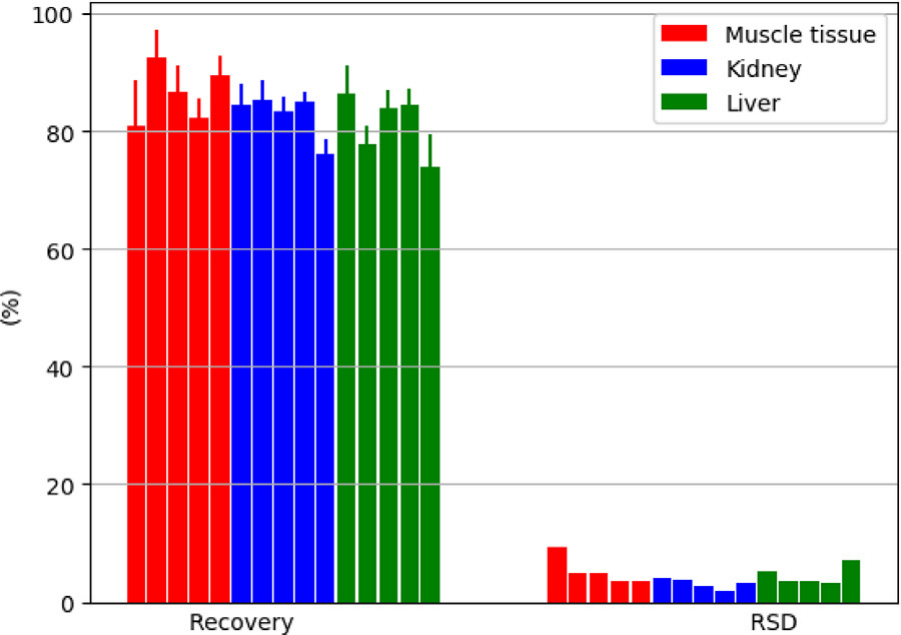
Reusability of MISPE cartridges for BPA determination in muscle tissue, kidney and liver, represented by recovery ± SD and RSD at a fortification level of 10 µg/kg (*n* = 2‒5 replicates/occasion) over 5 consecutive use cycles per animal matrix.

Five consecutive uses of MISPE BPA cartridges did not significantly impair the recovery rate or repeatability of BPA determination in all three matrices investigated in this study (Fig. 7). This finding is in line with the reported possible multiple use of MISPE for ochratoxin A determination in muscle tissue, kidney and liver [20], and significantly alleviates the impact of the analytical process using MIP technology from both environmental and economic aspects. However, at the fifth cycle the cartridges were blocked by the liver extracts, so a vacuum was needed at the SPE step to enable a sufficient flow rate. It is presumed, although has not been checked, that MISPE BPA cartridges could be used even more than five times for muscle tissue clean-up.

## Conclusions

In the present study the critical factors affecting the SPE step within the free BPA tissue determination, were systematically optimized using fortified sheep muscle tissue, kidney and liver. The use of a single MISPE purification step allied with HPLC-FLD gave relevant validation parameters considering recovery, precision, linearity and reporting levels. The analytical method used also proved to be applicable for tissues of other food-producing animals, and can serve as a basis for extended analysis of the total BPA (after enzymatic hydrolysis). Moreover, possible multiple use of the cartridges, with up to five re-use cycles, was proved with no significant loss in performance characteristics of the MISPE sorbent material for testing of BPA in the matrices investigated.

## Declaration of Competing Interests

The authors declare that they have no conflict of interest.

## Appendix Supplementary materials

Image, application 1
